# Quadruple Non-Insulin Therapy for Advanced Type 2 Diabetes Mellitus With Cognitive Impairment: A Case Report

**DOI:** 10.7759/cureus.108113

**Published:** 2026-05-01

**Authors:** Stjepan Skudar

**Affiliations:** 1 Family Medicine, Zagreb West Health Center, Zagreb, HRV

**Keywords:** diabetes type 2, family medicine practice, mild cognitive impairment (mci), prevention of diabetes, primary healthcare centers, primary medical care

## Abstract

Type 2 diabetes mellitus (T2DM) is a progressive metabolic disorder that often requires treatment intensification over time. In complex patients with multiple comorbidities and cognitive impairment, therapeutic decisions must balance glycemic control with safety and feasibility. We present a 59-year-old male with long-standing T2DM, marked body weight fluctuations, and emerging neurocognitive decline. Baseline glycated hemoglobin (HbA1c) was 9.4%, with progressive improvement to 7.1% under quadruple non-insulin therapy consisting of metformin, sitagliptin, glimepiride, and semaglutide. Insulin therapy was avoided due to cognitive impairment and a high risk of dosing errors. Sustained glycemic control was achieved without severe hypoglycemia. This case highlights the importance of individualized, multidisciplinary management in advanced T2DM and demonstrates that non-insulin strategies may be a viable and safe alternative in selected high-risk patients.

## Introduction

Type 2 diabetes mellitus (T2DM) is characterized by progressive insulin resistance and declining beta-cell function, often necessitating treatment intensification [1]. Although insulin therapy represents a cornerstone in advanced disease, its implementation may be limited in patients with cognitive impairment, substantial variability in body weight, and complex comorbidity profiles [2,3]. Recent recommendations from major diabetes associations emphasize individualized treatment approaches, particularly in vulnerable populations, where treatment burden and safety must be carefully balanced against glycemic targets [1].

While the use of multiple glucose-lowering agents is not uncommon in advanced type 2 diabetes, the clinical relevance of this case lies in the complexity of treatment decision-making in the context of cognitive impairment. In this patient, standard therapeutic strategies, including treatment intensification and medication optimization, were limited by tolerability issues and patient-related factors affecting adherence. This resulted in a non-standard therapeutic approach, highlighting the challenges of balancing glycemic control, safety, and treatment feasibility in real-world clinical practice. Therefore, this case is presented not for the rarity of the pharmacological combination itself but for its educational value in illustrating individualized diabetes management under complex clinical constraints.

## Case presentation

A 59-year-old male with T2DM diagnosed in 2015 was followed for progressive metabolic dysregulation. His clinical course was characterized by pronounced body weight fluctuations, with a peak weight of 118 kg corresponding to a body mass index (BMI) of 36.4 kg/m², followed by a reduction to 88 kg (BMI: 27.2 kg/m²), and a subsequent increase to 100 kg (BMI: 30.9 kg/m²) at the time of the most recent evaluation. At baseline, glycated hemoglobin (HbA1c) was 9.4%, reflecting poor glycemic control. Over time, pharmacological therapy was intensified in a stepwise manner. The patient had previously been treated with metformin-based therapy and short-term initiation of pioglitazone, which was discontinued shortly after introduction due to poor tolerability. In addition, the patient was not adherent to pioglitazone therapy and ultimately declined its continuation. Further escalation of GLP-1 receptor agonist therapy to semaglutide 2 mg weekly was proposed; however, the patient declined dose intensification due to concerns regarding treatment burden and injection frequency. The final treatment regimen consisted of metformin administered at a dose of 1000 mg twice daily, sitagliptin 100 mg once daily, glimepiride 4 mg once daily, and semaglutide, which was initially introduced as an oral formulation at 7 mg daily and later transitioned to a subcutaneous formulation with dose escalation to 1.0 mg weekly [4].

Following optimization of the therapeutic regimen, a progressive and clinically meaningful improvement in glycemic control was observed (Table 1). After the transition to subcutaneous semaglutide in 2023, HbA1c decreased from 7.8% to 7.1% in 2024 following dose escalation, with further stabilization at 7.0% in 2025 and 6.9% in 2026. Overall, this reflects a sustained reduction achieved over approximately one to two years. Importantly, improved glycemic control was maintained without documented episodes of severe hypoglycemia and without the need for insulin therapy.

**Table 1:** Timeline of therapeutic interventions and glycemic control Reference range for HbA1c: 4.0%-5.6% (normal), 5.7%-6.4% (prediabetes), and ≥6.5% (diabetes).

Year	Clinical Event/Intervention	HbA1c (%)
2015	Diagnosis of T2DM; metformin initiated	8.2
2019	Addition of sitagliptin	8.7
2021	Addition of glimepiride	9.1
2022	Initiation of oral semaglutide	9.4
2023	Transition to subcutaneous semaglutide	7.8
2024	Dose escalation of semaglutide	7.1
2025	Maintenance of quadruple therapy; stable weight and glycemic control	7.0
2026	Continued stability without insulin; no severe hypoglycemia	6.9

**Figure 1:**
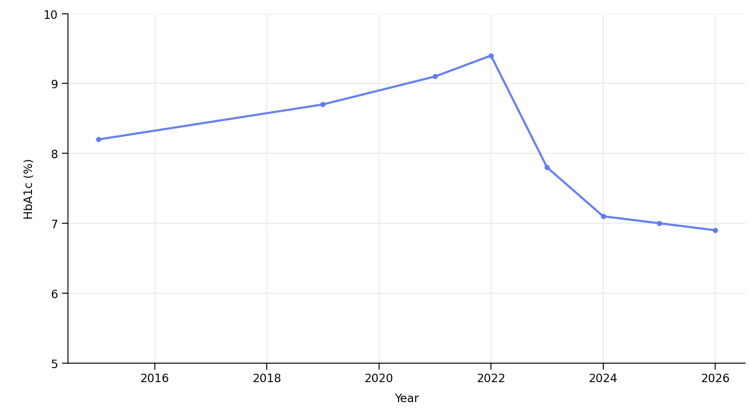
Trend of HbA1c over time

As shown in Figure 1, the progression of HbA1c values over time closely follows the sequence of therapeutic interventions. A gradual worsening of glycemic control is evident until 2022, followed by a marked and sustained decline after the initiation and subsequent escalation of semaglutide, particularly after the transition to the subcutaneous formulation. This stepwise intensification resulted in a reduction of HbA1c from a peak of 9.4% to 7.1% in 2024, with further stabilization at near-target levels (≤7.0%) in subsequent years. Notably, these improvements were achieved without the need for insulin therapy and in the absence of severe hypoglycemic episodes, illustrating the transition from suboptimal to well-controlled glycemia.

The patient’s medical history was notable for multiple comorbidities, including paroxysmal atrial fibrillation, dyslipidemia, a chronic liver lesion of unclear etiology, and degenerative musculoskeletal disease. His surgical history included colorectal tumor resection, total hip arthroplasty, and knee arthroscopy. A long-standing history of alcohol consumption was also present. During follow-up, the patient developed progressive neurocognitive impairment, manifesting as deficits in memory, attention, and executive function, along with disorientation in everyday situations and gait instability. Clinically notable behaviors included misplacement of personal belongings in inappropriate locations and difficulty recognizing familiar names. Neuropsychological testing using the Montreal Cognitive Assessment (MoCA) yielded a score of 21 out of 30, consistent with mild-to-moderate cognitive impairment [5]. Further diagnostic evaluation was initiated to assess potential reversible causes of cognitive decline, including laboratory testing of vitamin B12, vitamin D, folate, and thyroid function, as well as brain magnetic resonance imaging. The laboratory findings are summarized in Table 2. Vitamin B12, folate, and thyroid function parameters were within normal limits, while vitamin D levels were mildly reduced, without a clear causal relationship to the patient’s cognitive symptoms. Given the progression of neurocognitive impairment, neuroradiological evaluation was indicated, and brain magnetic resonance imaging (MRI) was scheduled to assess for structural or neurodegenerative changes. Considering the patient’s cognitive decline and the associated risk of incorrect medication administration, particularly with complex dosing regimens, initiation of insulin therapy was deemed unsafe and therefore deferred [1].

**Table 2:** Laboratory investigations

Parameter	Patient Value	Reference Range
Vitamin B12	412 pg/mL	200–900 pg/mL
Vitamin D (25-OH)	24 ng/mL	30–100 ng/mL
Folate	8.6 ng/mL	3–17 ng/mL
TSH	2.1 mIU/L	0.4–4.0 mIU/L
Free T4 (FT4)	1.2 ng/dL	0.8–1.8 ng/dL
Free T3 (FT3)	3.1 pg/mL	2.0–4.4 pg/mL

## Discussion

This case illustrates the complexity of managing advanced T2DM in a patient with significant metabolic variability and cognitive impairment [2]. The observed reduction in HbA1c from 9.4% to 7.1% demonstrates that a carefully tailored non-insulin therapeutic strategy can achieve meaningful metabolic improvement. Metformin provided a foundation by reducing hepatic glucose production and improving insulin sensitivity [1], while sitagliptin enhanced incretin-mediated insulin secretion [6]. Glimepiride contributed additional insulinotropic effects but required cautious use due to the risk of hypoglycemia. Semaglutide played a central role, offering potent glucose-lowering efficacy, weight reduction, and favorable cardiometabolic effects [7,8]. Although current guidelines generally discourage the combined use of GLP-1 receptor agonists and DPP-4 inhibitors due to overlapping mechanisms, their use in this case reflects individualized clinical decision-making in a context of limited therapeutic options and complex patient-specific factors [1,9]. Importantly, contemporary guidelines from the American Diabetes Association emphasize the need to individualize glycemic targets and treatment strategies, particularly in patients with comorbidities and cognitive impairment [10]. In this patient, cognitive decline significantly influenced therapeutic decisions, as the risk of insulin mismanagement and associated hypoglycemia was considered substantial.

It is important to emphasize that this report reflects a single clinical case and does not establish a standard treatment approach. Previous treatment with pioglitazone was initiated but discontinued shortly after introduction due to poor tolerability, and the patient did not continue its use. This, together with suboptimal glycemic control on existing therapy, prompted consideration of further intensification. Escalation of semaglutide to 2 mg weekly was proposed; however, the patient declined dose intensification due to concerns regarding treatment burden and injection frequency, which may have impacted adherence [9]. In this context, an individualized therapeutic approach was adopted based on patient preferences, tolerability, and available treatment options at that time, including the addition of a DPP-4 inhibitor. However, it is important to emphasize that the combination of a DPP-4 inhibitor and a GLP-1 receptor agonist is generally not recommended in current clinical guidelines due to overlapping mechanisms of action and limited additional glycemic benefit. Furthermore, both drug classes have been associated with pancreatitis, and their combined use may theoretically increase this risk [1]. For this reason, such combinations should be used with caution and are not routinely advised in clinical practice. Alternative guideline-supported strategies, such as optimization of GLP-1 receptor agonist therapy or the use of other agents with different mechanisms of action, including acarbose or pioglitazone, may represent more appropriate therapeutic options in similar clinical scenarios [1].

## Conclusions

In patients with advanced T2DM and complex clinical profiles, treatment success depends not only on glycemic targets but also on safety, cognitive status, and quality of life. This case demonstrates that glycemic control can be achieved with a quadruple non-insulin regimen in a selected patient. However, this approach should be interpreted with caution, as it is not routinely recommended in clinical practice and may carry potential safety concerns. Individualized treatment decisions and adherence to current clinical guidelines remain essential.
